# Cartilage icing and chondrocalcinosis on knee radiographs in the differentiation between gout and calcium pyrophosphate deposition

**DOI:** 10.1371/journal.pone.0231508

**Published:** 2020-04-16

**Authors:** Anna L. Falkowski, Jon A. Jacobson, Vivek Kalia, Nathaniel B. Meyer, Girish Gandikota, Matheos Yosef, Ralf G. Thiele

**Affiliations:** 1 Department of Radiology, University of Michigan, Ann Arbor, Michigan, United States of America; 2 Department of Radiology, University Hospital Basel, University of Basel, Basel, Switzerland; 3 Michigan Institute for Clinical & Health Research (MICHR), Ann Arbor, MI, United States of America; 4 Department of Rheumatology, University of Rochester, Rochester, New York, United States of America; Johns Hopkins School of Medicine, UNITED STATES

## Abstract

**Objective:**

To determine if findings of “cartilage icing" and chondrocalcinosis on knee radiography can differentiate between gout and calcium pyrophosphate deposition (CPPD).

**Methods:**

IRB-approval was obtained and informed consent was waived for this retrospective study. Electronic medical records from over 2.3 million patients were searched for keywords to identify subjects with knee aspiration-proven cases of gout or CPPD. Radiographs were reviewed by two fellowship-trained musculoskeletal radiologists in randomized order, blinded to the patients’ diagnoses. Images were evaluated regarding the presence or absence of cartilage icing, chondrocalcinosis, tophi, gastrocnemius tendon calcification, and joint effusion. Descriptive statistics, sensitivity, specificity, positive and negative predictive values, and accuracy were calculated.

**Results:**

From 49 knee radiographic studies in 46 subjects (31 males and 15 females; mean age 66±13 years), 39% (19/49) showed gout and 61% (30/49) CPPD on aspiration. On knee radiographs, cartilage icing showed a higher sensitivity for CPPD than gout (53–67% and 26%, respectively). Chondrocalcinosis also showed a higher sensitivity for CPPD than gout (50–57% versus 5%), with 95% specificity and 94% positive predictive value for diagnosis of CPPD versus gout. Soft tissue tophus-like opacities were present in gout at the patellar tendon (5%, 1/19) and at the popliteus groove in CPPD (15%, 4/27). Gastrocnemius tendon calcification was present in 30% (8/27) of subjects with CPPD, and 5% (1/19) of gout.

**Conclusion:**

In subjects with joint aspiration-proven crystal disease of the knee, the radiographic finding of cartilage icing was seen in both gout and CPPD. Chondrocalcinosis (overall and hyaline cartilage) as well as gastrocnemius tendon calcification positively correlated with the diagnosis of CPPD over gout.

## Introduction

Gout and calcium pyrophosphate deposition (CPPD) can both cause arthropathies from deposition of monosodium urate or calcium pyrophosphate crystals, respectively [1, 2]. An accurate diagnosis is essential for treatment and prevention of disease progression [3, 4]. Because the clinical presentation of both crystal-related arthropathies may overlap, radiography is regularly obtained for an initial assessment. Although, EULAR (European League Against Rheumatism) recommendations are more in favor of ultrasound [5, 6]. If there were clues on the initial radiograph that could reliably differentiate these entities, such clues would help direct clinical management.

The findings of CPPD on knee radiography have been described and include calcification of the hyaline and fibrocartilage (or chondrocalcinosis), as well as periarticular calcification including the proximal gastrocnemius tendon [1, 7]. The radiographic features of gout have also been described and include tophi and typical erosions [1]. An additional finding of gout using ultrasound and/or dual-energy CT (DECT) is termed the “double contour sign,” where the deposition of monosodium urate crystals on the surface of the hyaline cartilage produces the impression of an additional contour [8–10].

Linear opacities on knee radiographs that outline the meniscus and hyaline cartilage have been shown in the literature [11, 12]. These opacities resemble an arthrogram effect and are opposed to amorphous punctate calcifications within hyaline cartilage and menisci as seen with chondrocalcinosis. This arthrogram appearance of linear opacities on radiography may be the equivalent of the double contour sign seen on ultrasound, and has recently been described in a case report on radiography in the diagnosis of gout with resolving of the sign after treatment [13]. In our study we will refer to this radiograph sign as “cartilage icing”, to differentiate it from the sonographic “double contour sign”. The purpose of the study was to determine if findings of cartilage icing and chondrocalcinosis on knee radiography can differentiate between gout and CPPD in cases of proven diagnosis.

## Materials and methods

### Inclusion and exclusion criteria

Institutional Review Board approval was obtained by the Medical School Institutional Review Board (IRBMED) of the University of Michigan for this retrospective study (HUM00142554), and informed consent was waived for this retrospective study. The Electronic Medical Record Search Engine (EMERSE) [14] was used to search a database of over 2.3 million patients. This database consists of all electronic medical records of patients’, including patients’ history, referrals, laboratory values, radiologic reports, etc. The database was searched for key words: gout (n = 86,482), calcium pyrophosphate deposition disease, CPPD, pseudogout (n = 9,243), knee radiographs (n = 1,048 with term gout in patient's documents / n = 355 with term CPPD, etc. in patient's documents, respectively) and joint aspiration (n = 209 / n = 119, respectively), arthrocentesis (n = 544 / n = 241, respectively). Merging of all search groups created a number of patients of 634. However, negations of the terms, i.e. subjects without any knee radiographs and without aspiration-proven cases of gout or CPPD (e.g. aspiration performed to exclude infection, without crystal analysis) were excluded (n = 563). Patients were also excluded with no obtained radiographs of the aspirated knee (n = 4), joint aspiration findings of both gout and CPPD (n = 5), as well as those post-operative (n = 7). Patients with joint aspiration proof of CPPD and either elevated serum urate or history of colchicine or allopurinol use were excluded to avoid subjects with both CPPD and gout (n = 9).

### Radiographic review

**Fig 1:**
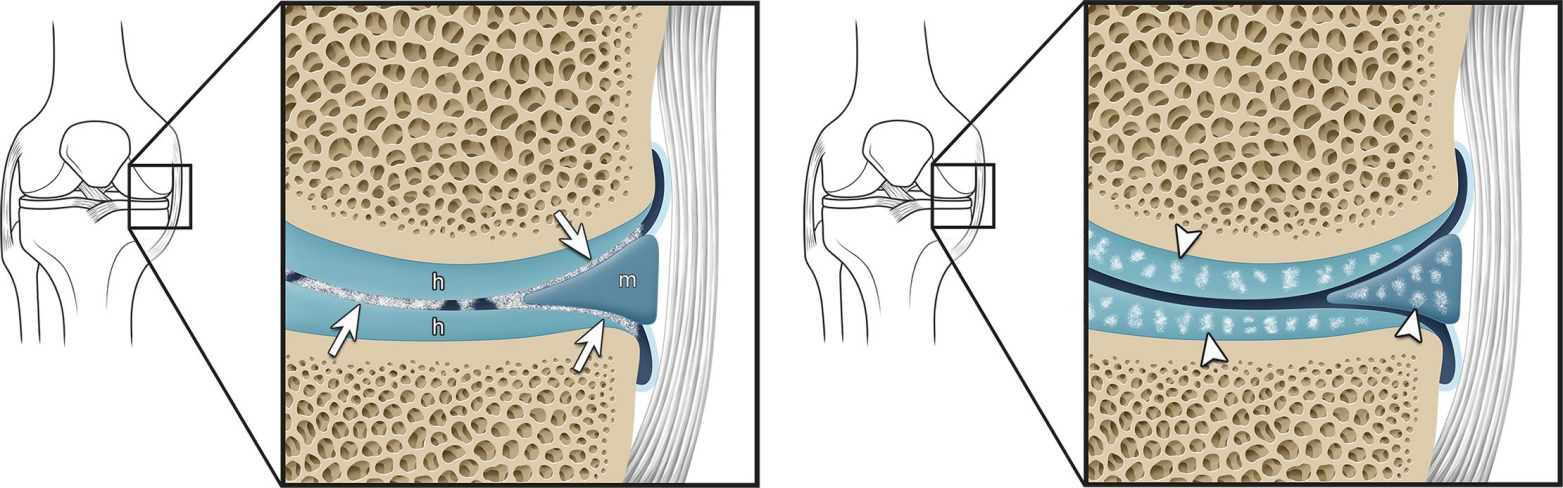
Illustrations show (A) cartilage icing (arrows) and (B) chondrocalcinosis (arrowheads).

Anteroposterior and lateral knee radiographs of the study group were retrospectively reviewed in randomized order by two fellowship-trained musculoskeletal radiologists (23 and 2 years of experience). Both readers were blinded to the subjects’ diagnoses. Radiographs were independently evaluated by the two readers for the findings of linear density outlining the cartilage as an arthrogram effect (cartilage icing) or the presence of punctate density or calcification within the cartilage (chondrocalcinosis) (Fig 1). The locations of the above findings on the anteroposterior radiograph were recorded as medial meniscus, medial compartment hyaline cartilage, lateral meniscus, and lateral compartment hyaline cartilage. The locations of the above findings on the lateral radiograph were recorded as anterior meniscus, anterior hyaline cartilage, posterior meniscus, and posterior hyaline cartilage.

Additional radiographic findings were also recorded by consensus, which included the presence of tophus formation (appearing as an amorphous soft tissue density) at the popliteal groove (anteroposterior view), patellar tendon (lateral view), or other sites (any view). Calcifications of the gastrocnemius tendon on lateral views were also recorded. For each of the findings assessed, a note was made if the finding was definitely present, definitely absent, indeterminate, or could not be assessed. Only data points where the finding was definitely present or absent were included in the final analysis. Presence of osteoarthritis was documented for the medial and lateral femorotibial compartment, as well as the patellofemoral compartment using the Kellgren-Lawrence-score [15]. Also, the presence of suprapatellar recess distention (if measuring greater than 5 mm anteroposterior) on lateral knee radiographs was recorded, including measurements. Lastly, correlation was made to ultrasound and dual-energy CT if present, as well as other clinical and laboratory information.

### Statistical analysis

Descriptive statistics (including mean, standard deviation, range, and percentage), sensitivity, specificity, positive and negative predictive value, and accuracy were used for data evaluation. Cohen’s Kappa as well as prevalence-adjusted bias-adjusted kappa (PABAK) statistics for inter-reader reliability were calculated. PABAK was necessitated in situations where prevalence of a response is very high or very low resulting in a paradox of low kappa even though the proportion of agreements between the readers is high [16].

## Results

### Database search

**Fig 2:**
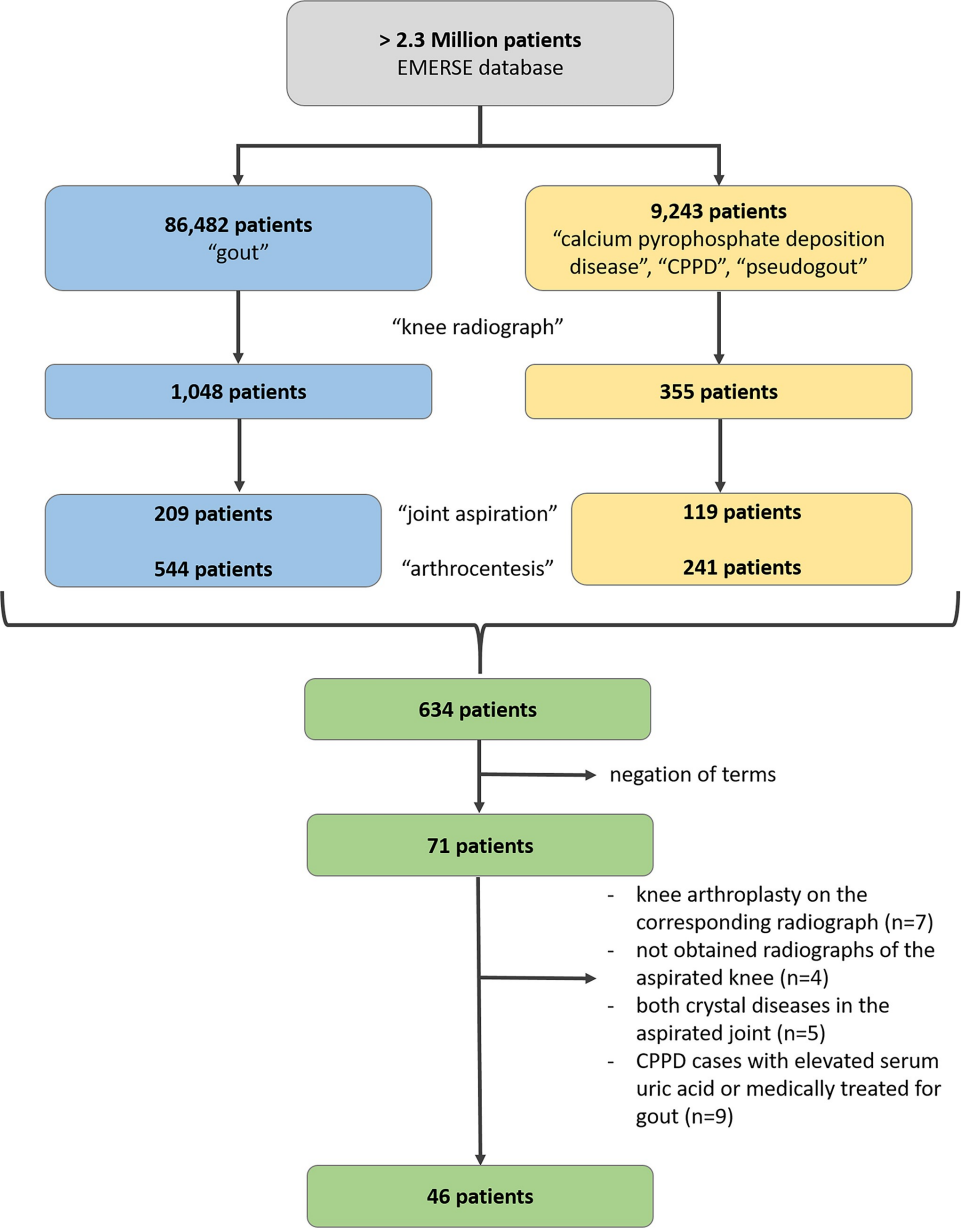
Chart shows the key word search for patients with gout or CPPD having a knee radiograph with a joint aspiration prove of the diagnosis of the same joint. Blue color is the search for gout, yellow color is the search for CPPD, and green color is after merging all of the patients. The number after merging declined, due to overlap of patients in both groups.

**Fig 3:**
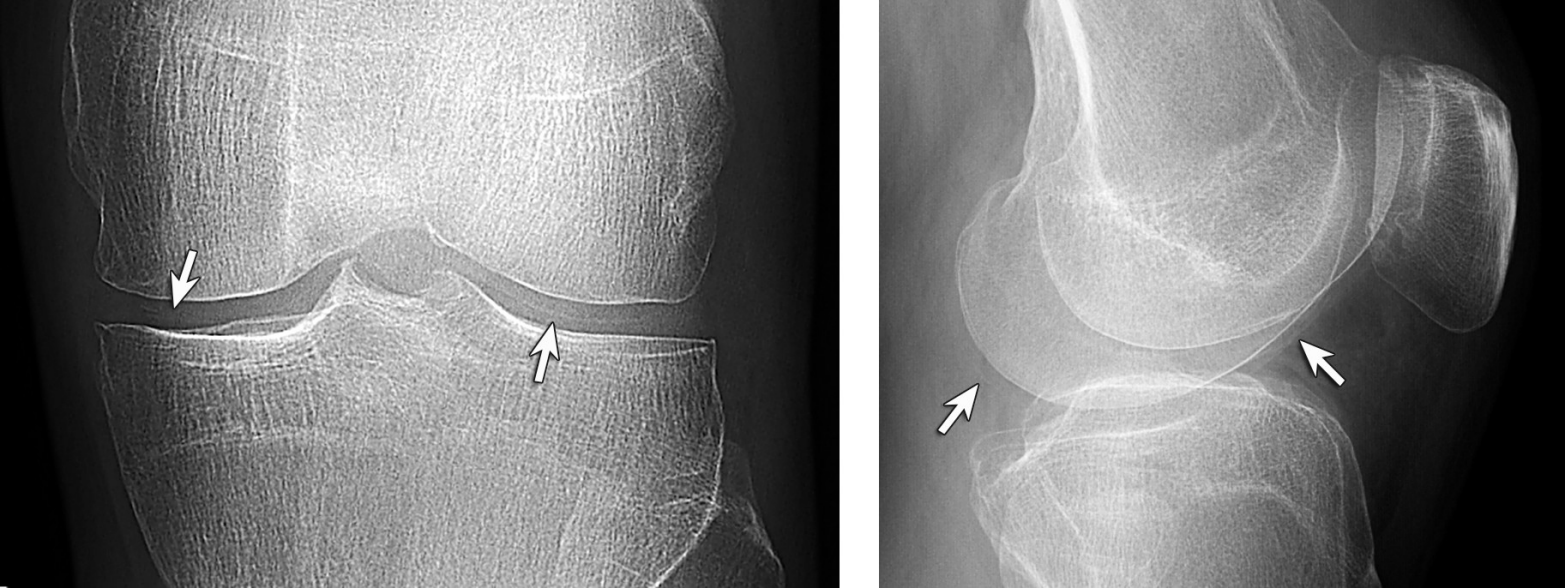
40-year-old man with gout. Knee radiographs (A, anteroposterior and B, lateral) show linear cartilage icing (arrows). No chondrocalcinosis is present on the images.

**Fig 4:**
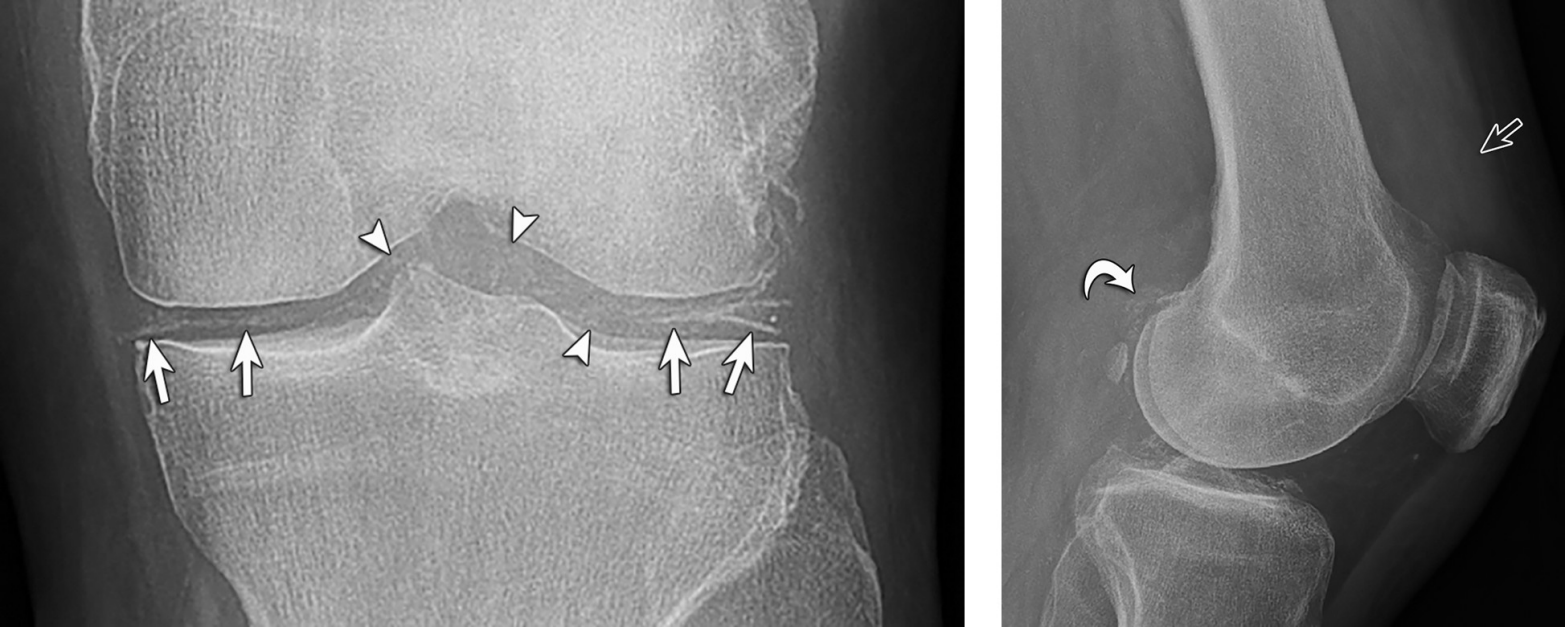
66-year-old man with CPPD. (**A**) Anteroposterior knee radiograph shows cartilage icing with linear opacity outlining of the menisci and hyaline cartilage (arrows). Chondrocalcinosis appears as speckled calcifications within the hyaline cartilage (arrowheads). (**B**) Lateral knee radiograph shows calcifications of the gastrocnemius tendon (curved arrow) and distention of the suprapatellar recess (open arrow) characterized as joint effusion.

The final study group consisted of 46 patients (Fig 2). Three of these subjects had bilateral knee involvement (all three patients had CPPD); therefore, there were 49 knee radiographic studies for review. Of the 49 knee radiographs from 46 subjects, 39% (19/49) had aspiration-proven gout (Fig 3) and 61% (30/49) had aspiration-proven CPPD (Fig 4).

### Demographics

Of the 46 subjects, 67% were male (31/46) and 33% female (15/46). The male:female ratio was 84%:16% in the gout group and 40%:60% in the CPPD group. The average age of the patients was 65 years (range 37 to 88 years; standard deviation 12 years). In the subjects with gout, the average age was 57 years (range 37 to 81 years; standard deviation 12 years), and in the CPPD subjects 70 years (range 54 to 88 years; standard deviation 9 years). The radiographs of the knee were performed between April 2001 and June 2018. The time interval between radiograph and joint aspiration was a mean of 61 days. The number of patients with a knee radiograph and joint aspiration of the same joint within a time interval of 4 days was 32 patients. Of the 46 total subjects, 9 also had ultrasound and none had dual-energy or multi-energy CT. Of the subjects with joint-aspiration proven gout, 89% (17/19) had serum urate levels available (mean 7.6 mg/dl and range 3.7–11.4 mg/dl), where levels were abnormally elevated in 47% (8/17) (greater than 6.0 mg/dl in females and 7.8 mg/dl in males according to our institutional laboratory), and 26% (5/19) were being medically treated for gout. Of the subjects with joint-aspiration proven CPPD 74% (20/27) had serum urate levels available (mean 5.6 mg/dl and range 4.1–7.7 mg/dl).

### Radiographic findings

#### Cartilage icing

The results of cartilage icing in subjects with gout (Fig 3) and CPPD (Fig 4) at each anatomic location are listed on Tables 1 and 2 for each reader. The inter-reader reliability for cartilage icing at the anteroposterior knee radiographs were for Cohen’s Kappa moderate to substantial (Ƙ = 0.5194–0.7241) and for PABAK moderate to almost perfect (P-B adjusted Ƙ = 0.5909–0.8286). For lateral radiographs the Kappa values were fair to substantial (Ƙ = 0.0563–0.6538), and substantial to almost perfect (P-B adjusted Ƙ = 0.7436–0.9444), respectively. Cartilage icing was more frequently identified on the anteroposterior radiographs (47% or 23/49) than lateral radiographs (14% or 7/49).

**Table 1:** The results for sensitivity, specificity, positive predictive value, negative predictive value, and accuracy for cartilage icing differentiating gout from CPPD for each reader.

CARTILAGE ICING				Gout vs. CPPD									
				Sensitivity	95% CI	Specificity	95% CI	PPV	95% CI	NPV	95% CI	Accuracy	95% CI
AP Radio-graph	Medial	Meniscus	Reader 1	17% (3/18)	0–34%	56% (15/27)	37–74%	20% (3/15)	0–40%	50% (15/30)	32–68%	40% (18/45)	26–54%
			Reader 2	11%(2/19)	0–24%	58% (15/26)	39–77%	15% (2/13)	0–35%	47% (15/32)	30–64%	38% (17/45)	24–52%
		Hyaline Cartilage	Reader 1	11% (2/18)	0–26%	96% (24/25)	88–100%	67% (2/3)	13–100%	60% (24/40)	45–75%	60% (26/43)	46–75%
			Reader 2	12% (2/17)	0–27%	79% (15/19)	61–97%	33% (2/6)	0–71%	50% (15/30)	32–68%	47% (17/36)	31–64%
	Lateral	Meniscus	Reader 1	16% (3/19)	0–32%	36% (8/22)	16–56%	18% (3/17)	0–36%	33% (8/24)	14–52%	27% (11/41)	13–40%
			Reader 2	21% (4/19)	3–39%	43% (10/23)	23–64%	24% (4/17)	3–44%	40% (10/25)	21–59%	33% (14/42)	19–48%
		Hyaline Cartilage	Reader 1	16% (3/19)	0–32%	67% (16/24)	48–86%	27% (3/11)	1–54%	50% (16/32)	33–67%	44% (19/43)	29–59%
			Reader 2	16% (3/19)	0–32%	53% (9/17)	29–77%	27% (3/11)	1–54%	36% (9/25)	17–55%	33% (12/36)	18–49%
Lateral Radio-graph	Anterior	Meniscus	Reader 1	11% (2/18)	0–26%	83% (20/24)	68–98%	33% (2/6)	0–71%	56% (20/36)	39–72%	52% (22/42)	37–67%
			Reader 2	0% (0/19)	0–0%	95% (21/22)	87–100%	0% (0/1)	0–0%	53% (21/40)	37–68%	51% (21/41)	36–67%
		Hyaline Cartilage	Reader 1	22% (4/18)	3–41%	93% (27/29)	84–100%	67% (4/6)	29–100%	66% (27/41)	51–80%	66% (31/47)	52–80%
			Reader 2	6% (1/17)	0–17%	96% (27/28)	90–100%	50% (1/2)	0–100%	63% (27/43)	48–77%	62% (28/45)	48–76%
	Posterior	Meniscus	Reader 1	0% (0/19)	0–0%	90% (18/20)	77–100%	0%(0/2)	0–0%	49% (18/37)	33–65%	46% (18/39)	31–62%
			Reader 2	0% (0/19)	0–0%	95% (20/21)	86–100%	0% (0/1)	0–0%	51% (20/39)	36–67%	50% (20/40)	35–65%
		Hyaline Cartilage	Reader 1	16% (3/19)	0–32%	100% (29/29)	100–100%	100% (3/3)	100–100%	64% (29/45)	50–78%	67% (32/48)	53–80%
			Reader 2	5% (1/19)	0–15%	96% (25/26)	89–100%	50% (1/2)	0–100%	58% (25/43)	43–73%	58% (26/45)	43–72%
Any Radio-graphic View		Any Cartilage and Location	Reader 1	26% (5/19)	7–46%	33% (10/30)	16–50%	20% (5/25)	4–36%	42% (10/24)	22–61%	31%(15/49)	18–44%
			Reader 2	26% (5/19)	7–46%	47% (14/30)	29–65%	24% (5/21)	6–42%	50% (14/28)	31–69%	39%(19/49)	25–52%

PPV, positive predictive value; NPV, negative predictive value; AP, anteroposterior

**Table 2:** The results for sensitivity, specificity, positive predictive value, negative predictive value, and accuracy for cartilage icing differentiating CPPD from gout for each reader.

CARTILAGE ICING				CPPD vs. Gout									
				Sensitivity	95% CI	Specificity	95% CI	PPV	95% CI	NPV	95% CI	Accuracy	95% CI
AP Radio-graph	Medial	Meniscus	Reader 1	44% (12/27)	26–63%	83% (15/18)	66–100%	80% (12/15)	60–100%	50% (15/30)	32–68%	60% (27/45)	46–74%
			Reader 2	42% (11/26)	23–61%	89% (17/19)	76–100%	85% (11/13)	65–100%	53% (17/32)	36–70%	62% (28/45)	48–76%
		Hyaline Cartilage	Reader 1	4% (1/25)	0–12%	89% (16/18)	74–100%	33% (1/3)	0–87%	40% (16/40)	25–55%	40% (17/43)	25–54%
			Reader 2	21% (4/19)	3–39%	88% (15/17)	73–100%	67% (4/6)	29–100%	50% (15/30)	32–68%	53% (19/36)	36–69%
	Lateral	Meniscus	Reader 1	64% (14/22)	44–84%	84% (16/19)	68–100%	82% (14/17)	64–100%	67% (16/24)	48–86%	73% (30/41)	60–87%
			Reader 2	57% (13/23)	36–77%	79% (15/19)	61–97%	76% (13/17)	56–97%	60% (15/25)	41–79%	67% (28/42)	52–81%
		Hyaline Cartilage	Reader 1	33% (8/24)	14–52%	84% (16/19)	68–100%	73% (8/11)	46–99%	50% (16/32)	33–67%	56% (24/43)	41–71%
			Reader 2	47%(8/17)	23–71%	84%(16/19)	68–100%	73% (8/11)	46–99%	64% (16/25)	45–83%	67% (24/36)	51–82%
Lateral Radio-graph	Anterior	Meniscus	Reader 1	17% (4/24)	2–32%	89% (16/18)	74–100%	67% (4/6)	29–100%	44% (16/36)	28–61%	48% (20/42)	33–63%
			Reader 2	5% (1/22)	0–13%	100% (19/19)	100–100%	100% (1/1)	100–100%	48% (19/40)	32–63%	49% (20/41)	33–64%
		Hyaline Cartilage	Reader 1	7% (2/29)	0–16%	78% (14/18)	59–97%	33% (2/6)	0–71%	34% (14/41)	20–49%	34% (16/47)	20–48%
			Reader 2	4% (1/28)	0–10%	94% (16/17)	83–100%	50% (1/2)	0–100%	37% (16/43)	23–52%	38% (17/45)	24–52%
	Posterior	Meniscus	Reader 1	10% (2/20)	0–23%	100% (19/19)	100–100%	100% (2/2)	100–100%	51% (19/37)	35–67%	54% (21/39)	38–69%
			Reader 2	5%(1/21)	0–14%	100%(19/19)	100–100%	100% (1/1)	100–100%	49% (19/39)	33–64%	50% (20/40)	35–65%
		Hyaline Cartilage	Reader 1	0% (0/29)	0–0%	84% (16/19)	68–100%	0% (0/3)	0–0%	36% (16/45)	22–50%	33% (16/48)	20–47%
			Reader 2	4% (1/26)	0–11%	95% (18/19)	85–100%	50% (1/2)	0–100%	42% (18/43)	27–57%	42% (19/45)	28–57%
Any Radio-graphic View		Any Cartilage and Location	Reader 1	67% (20/30)	50–84%	74% (14/19)	54–93%	80% (20/25)	64–96%	58% (14/24)	39–78%	69% (34/49)	56–82%
			Reader 2	53% (16/30)	35–71%	74% (14/19)	54–93%	76% (16/21)	58–94%	50% (14/28)	31–69%	61% (30/49)	48–75%

PPV, positive predictive value; NPV, negative predictive value; AP, anteroposterior

#### Chondrocalcinosis

**Fig 5:**
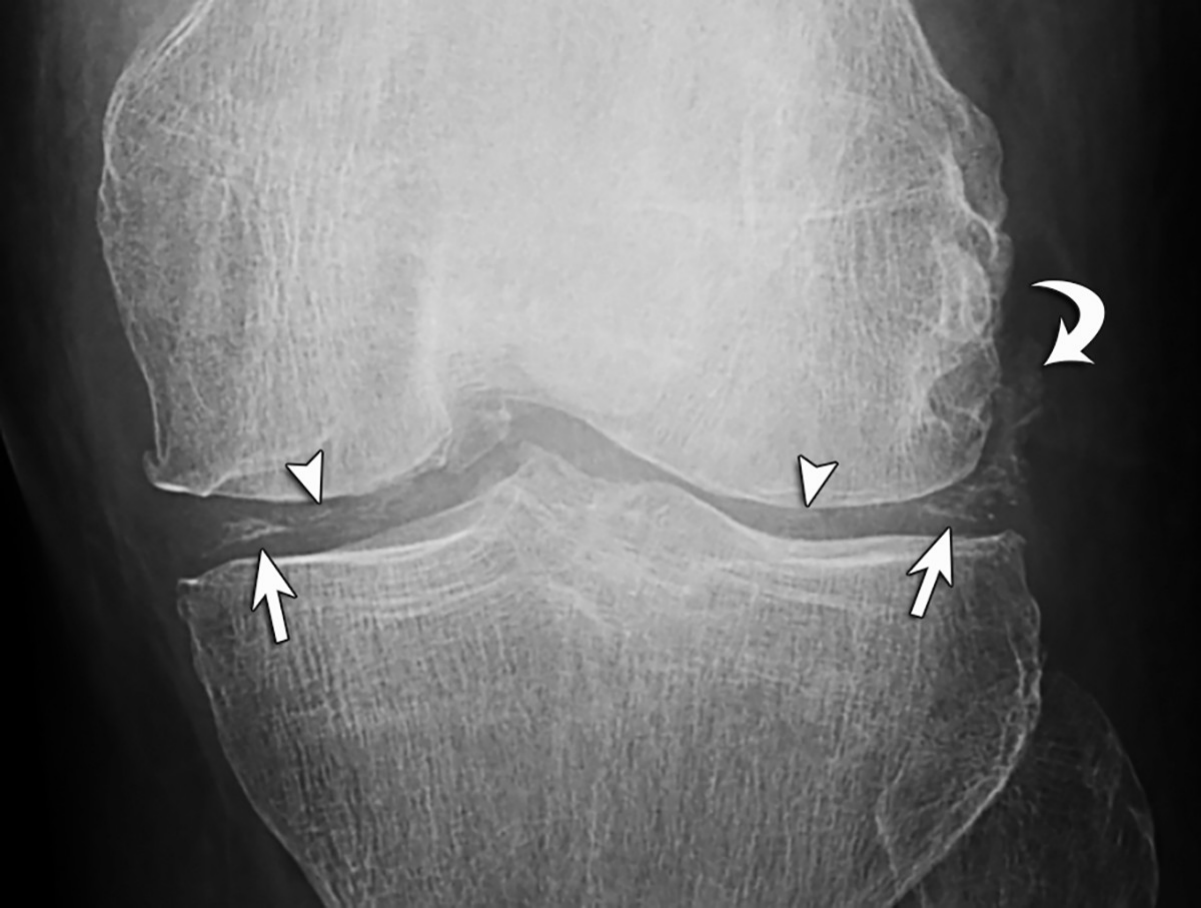
82-year-old woman with CPPD. Anteroposterior knee radiograph shows cartilage icing with linear opacity outlining of the menisci and hyaline cartilage (arrows). Chondrocalcinosis appears as speckled calcifications within the hyaline cartilage (arrowheads). Note amorphous opacity characterized as a tophus (curved arrow) at the popliteus groove.

The results of chondrocalcinosis in subjects with CPPD (Figs 4 and 5) versus gout at each anatomic location are listed in Tables 3 and 4 for each reader. The inter-reader reliability for chondrocalcinosis at the anteroposterior knee radiographs were for Cohen’s Kappa moderate to almost perfect (Ƙ = 0.4160–0.9416) and for PABAK moderate to almost perfect (P-B adjusted Ƙ = 0.5385–0.9556). For lateral radiographs the Kappa values were fair to almost perfect (Ƙ = 0.3684–1.0000), and substantial to almost perfect (P-B adjusted Ƙ. For lateral radiographs the Chondrocalcinosis was identified on anteroposterior radiographs in 26.5% (13/49) and on lateral radiographs in 24% (12/49).

**Table 3:** The results for sensitivity, specificity, positive predictive value, negative predictive value, and accuracy for chondrocalcinosis differentiating gout from CPPD for each reader.

CHONDROCALCINOSIS				Gout vs. CPPD									
				Sensitivity	95% CI	Specificity	95% CI	PPV	95% CI	NPV	95% CI	Accuracy	95% CI
AP Radio-graph	Medial	Meniscus	Reader 1	5% (1/19)	0–15%	59% (16/27)	41–78%	8% (1/12)	0–24%	47% (16/34)	30–64%	37% (17/46)	23–51%
			Reader 2	5% (1/19)	0–15%	62% (16/26)	43–80%	9%(1/11)	0–26%	47% (16/34)	30–64%	38% (17/45)	24–52%
		Hyaline Cartilage	Reader 1	6% (1/18)	0–16%	46% (11/24)	26–66%	7% (1/14)	0–21%	39% (11/28)	21–57%	29% (12/42)	15–42%
			Reader 2	0% (0/18)	0–0%	79%(19/24)	63–95%	0% (0/5)	0–0%	51% (19/37)	35–67%	45% (19/42)	30–60%
	Lateral	Meniscus	Reader 1	0% (0/18)	0–0%	36% (8/22)	16–56%	0% (0/14)	0–0%	31% (8/26)	13–49%	20% (8/40)	8–32%
			Reader 2	5% (1/19)	0–15%	42% (10/24)	22–61%	7% (1/15)	0–19%	36% (10/28)	18–53%	26% (11/43)	13–39%
		Hyaline Cartilage	Reader 1	6% (1/18)	0–16%	57% (13/23)	36–77%	9% (1/11)	0–26%	43% (13/30)	26–61%	34% (14/41)	20–49%
			Reader 2	5% (1/19)	0–15%	77% (17/22)	60–95%	17% (1/6)	0–46%	49% (17/35)	32–65%	44% (18/41)	29–59%
Lateral Radio-graph	Anterior	Meniscus	Reader 1	0% (0/19)	0–0%	70% (16/23)	51–88%	0% (0/7)	0–0%	46% (16/35)	29–62%	38% (16/42)	23–53%
			Reader 2	5% (1/19)	0–15%	65% (15/23)	46–85%	11% (1/9)	0–32%	45% (15/33)	28–62%	38% (16/42)	23–53%
		Hyaline Cartilage	Reader 1	0% (0/19)	0–0%	90% (26/29)	79–100%	0% (0/3)	0–0%	58% (26/45)	43–72%	54% (26/48)	40–68%
			Reader 2	0% (0/19)	0–0%	93% (27/29)	84–100%	0% (0/2)	0–0%	59% (27/46)	44–73%	56% (27/48)	42–70%
	Posterior	Meniscus	Reader 1	5% (1/19)	0–15%	61% (11/18)	39–84%	13% (1/8)	0–35%	38% (11/29)	20–56%	32% (12/37)	17–48%
			Reader 2	5% (1/19)	0–15%	50% (12/24)	30–70%	8% (1/13)	0–22%	40% (12/30)	22–58%	30% (13/43)	17–44%
		Hyaline Cartilage	Reader 1	0% (0/19)	0–0%	79% (23/29)	65–94%	0% (0/6)	0–0%	55% (23/42)	40–70%	48% (23/48)	34–62%
			Reader 2	0% (0/18)	0–0%	80% (20/25)	64–96%	0% (0/5)	0–0%	53% (20/38)	37–69%	47% (20/43)	32–61%
Any Radio-graphic View		Any Cartilage and Location	Reader 1	5% (1/19)	0–15%	43% (13/30)	26–61%	6% (1/18)	0–16%	42% (13/31)	25–59%	29% (14/49)	16–41%
			Reader 2	5% (1/19)	0–15%	50% (15/30)	32–68%	6% (1/16)	0–18%	45% (15/33)	28–62%	33% (16/49)	20–46%

PPV, positive predictive value; NPV, negative predictive value; AP, anteroposterior

**Table 4:** The results for sensitivity, specificity, positive predictive value, negative predictive value, and accuracy for chondrocalcinosis differentiating CPPD from gout for each reader.

CHONDROCALCINOSIS				CPPD vs. Gout									
				Sensitivity	95% CI	Specificity	95% CI	PPV	95% CI	NPV	95% CI	Accuracy	95% CI
AP Radio-graph	Medial	Meniscus	Reader 1	41% (11/27)	22–59%	95% (18/19)	85–100%	92% (11/12)	76–100%	53% (18/34)	36–70%	63% (29/46)	49–77%
			Reader 2	38% (10/26)	20–57%	95% (18/19)	85–100%	91% (10/11)	74–100%	53% (18/34)	36–70%	62% (28/45)	48–76%
		Hyaline Cartilage	Reader 1	54% (13/24)	34–74%	94% (17/18)	84–100%	93% (13/14)	79–100%	61% (17/28)	43–79%	71% (30/42)	58–85%
			Reader 2	21% (5/24)	5–37%	100% (18/18)	100–100%	100% (5/5)	100–100%	49% (18/37)	33–65%	55% (23/42)	40–70%
	Lateral	Meniscus	Reader 1	64% (14/22)	44–84%	100% (18/18)	100–100%	100% (14/14)	100–100%	69% (18/26)	51–87%	80% (32/40)	68–92%
			Reader 2	58% (14/24)	39–78%	95% (18/19)	85–100%	93% (14/15)	81–100%	64% (18/28)	47–82%	74% (32/43)	61–87%
		Hyaline Cartilage	Reader 1	43% (10/23)	23–64%	94% (17/18)	84–100%	91% (10/11)	74–100%	57% (17/30)	39–74%	66% (27/41)	51–80%
			Reader 2	23% (5/22)	5–40%	95% (18/19)	85–100%	83% (5/6)	54–100%	51% (18/35)	35–68%	56% (23/41)	41–71%
Lateral Radio-graph	Anterior	Meniscus	Reader 1	30% (7/23)	12–49%	100% (19/19)	100–100%	100% (7/7)	100–100%	54% (19/35)	38–71%	62% (26/42)	47–77%
			Reader 2	35% (8/23)	15–54%	95% (18/19)	85–100%	89% (8/9)	68–100%	55% (18/33)	38–72%	62% (26/42)	47–77%
		Hyaline Cartilage	Reader 1	10% (3/29)	0–21%	100% (19/19)	100–100%	100% (3/3)	100–100%	42% (19/45)	28–57%	46% (22/48)	32–60%
			Reader 2	7% (2/29)	0–16%	100% (19/19)	100–100%	100% (2/2)	100–100%	41% (19/46)	27–56%	44% (21/48)	30–58%
	Posterior	Meniscus	Reader 1	39% (7/18)	16–61%	95% (18/19)	85–100%	88% (7/8)	65–100%	62% (18/29)	44–80%	68% (25/37)	52–83%
			Reader 2	50% (12/24)	30–70%	95% (18/19)	85–100%	92% (12/13)	65–100%	60% (18/30)	42–78%	70% (30/43)	56–83%
		Hyaline Cartilage	Reader 1	21% (6/29)	6–35%	100% (19/19)	100–100%	100% (6/6)	100–100%	45% (19/42)	30–60%	52% (25/48)	38–66%
			Reader 2	20% (5/25)	4–36%	100% (18/18)	100–100%	100% (5/5)	100–100%	47% (18/38)	31–63%	53% (23/43)	39–68%
Any Radio-graphic View		Any Cartilage and Location	Reader 1	57% (17/30)	39–74%	95% (18/19)	85–100%	94% (17/18)	84–100%	58% (18/31)	41–75%	71% (35/49)	59–84%
			Reader 2	50% (15/30)	32–68%	95% (18/19)	85–100%	94% (15/16)	82–100%	55% (18/33)	38–72%	67% (33/49)	54–80%

PPV, positive predictive value; NPV, negative predictive value; AP, anteroposterior

#### Other radiographic findings

An amorphous soft tissue opacity, similar to a “tophus” described in gout, was identified at the proximal popliteus tendon on radiographs in no gout case and 15% (4/27) of CPPD patients (Fig 5). An amorphous tophus-like opacity was identified on radiography at the patellar tendon in 5% (1/19) of gout cases and in no CPPD cases. Amorphous tophus-like opacities at other periarticular sites were found in no gout and 7% (2/27) of CPPD patients.

Calcification of the gastrocnemius tendon on lateral radiographs (Fig 4B) was present in subjects with CPPD in 30% (8/27) of cases and absent in 70% (19/27). In subjects with gout, gastrocnemius tendon calcification was present in 5% (1/19), and absent in 95% (18/19).

With regard to osteoarthritis, subjects with gout showed in the medial compartment mostly none to mild osteoarthritis in an equal distribution of 32% (6/19), and in the lateral compartment mostly no osteoarthritis (53% or 10/19) on anteroposterior radiographs (according to Kellgren-Lawrence) [15]. On lateral radiographs, subjects with gout had mostly minimal patellofemoral osteoarthritis (58% or 11/19). In subjects with CPPD, the medial compartment showed mostly moderate osteoarthritis (41% or 11/27 cases) and mostly severe osteoarthritis at the lateral compartment (30% or 8/27). On lateral radiographs, patellofemoral osteoarthritis in patients with CPPD was present mostly in minimal (33% or 9/27) and severe (33% or 9/27) degree.

Joint effusion on radiographs was present in 100% (27/27) of subjects with CPPD (Fig 4B), measuring on average 14 mm in anteroposterior dimension at the suprapatellar recess, and in 79% (15/19) of subjects having gout with an average dimension of 15.5 mm.

## Discussion

In our retrospective study, we evaluated potential radiographic signs that may differentiate gout from CPPD, including chondrocalcinosis and cartilage icing. Our results showed that cartilage icing on radiography was more common in CPPD than gout (53–67% and 26% sensitivity, respectively). Chondrocalcinosis was more common in CPPD than gout (50–57% versus 5% sensitivity), with 95% specificity and 94% positive predictive value for diagnosis of CPPD versus gout. Calcification of the proximal gastrocnemius tendon was most common in CPPD in 30% (8/27) of subjects, and 5% (1/19) of gout.

### Cartilage icing

The double contour sign was described at ultrasound when monosodium urate crystal deposits created a linear echogenic interface on the surface of the hypoechoic hyaline cartilage that paralleled the deeper hyperechoic subchondral bone plate [8]. It is described as specific for gout [17] and included in the gout classification criteria by the American College of Rheumatology (ACR) / EULAR initiative [5, 18]. However, Löffler et al. [2] showed that the double contour sign predicted crystal-related joint disease but could not distinguish between the types of crystals present. To our knowledge, cartilage icing on radiography has not been previously evaluated in this context. In our study, cartilage icing on radiography was present in both CPPD and gout, and more common in CPPD (53–67% and 26% sensitivity, respectively). In addition, cartilage icing on radiography had a 76–80% positive predictive value for CPPD versus gout in comparison to only a 20–24% positive predictive value for gout versus CPPD.

Diagnostic problems of the double contour sign on ultrasound, regarding the diagnosis of gout or CPPD, have been reported in literature and may be explained in several ways. One explanation could relate to a diagnostic error. For example, layering of gout crystals on the cartilage surface could be misinterpreted as being located within cartilage when an adjacent joint effusion is present [2]; also, deposition of CPPD crystals within the cartilage may be continuous and linear simulating a double contour [2]. As another explanation, Filippucci et al. [9] theorized that ‘‘shedding” of the CPPD crystals into the joint space might lead to a subsequent deposition of crystals on the cartilage surface creating a double contour sign [19]. Additionally, Misra et al. found calcifications in chondrocalcinosis to be present ubiquitously within knee joints [20].

This last hypothesis may explain the results of our study where the double contour sign was more common in patients with CPPD. While we could not determine the severity of patient symptoms in this retrospective study, we presume that patients were symptomatic given that they had radiographic imaging and joint aspiration; therefore, CPPD crystal shedding or ubiquitous presence may be more likely in our symptomatic study population. A counterargument to the shedding theory is that 27% (8/30) of our CPPD patients had cartilage icing on radiography but without chondrocalcinosis. It would stand to reason that underlying chondrocalcinosis should be present in more subjects if this was the origin of the shedding of crystals into the joint space. Another point of consideration is that although the joint aspiration was positive for CPPD, the crystals outlining the cartilage might be crystals due to basic calcium phosphate (BCP) crystals, e.g. hydroxyapatite crystal deposition disease (HADD) [10, 21, 22]. However, their diagnosis in joint aspiration is limited, because they are even smaller than CPPD or monosodium urate crystals and not visible on light microscopy, except when the crystals form aggregates [10, 23]. It is also possible that ultrasound findings of the double contour sign simply cannot be applied to the finding of cartilage icing at radiography. For example, perhaps ultrasound has a higher sensitivity in detecting cartilage icing in gout compared with radiography, as monosodium urate crystals although opaque are not calcified and may be more difficult to visualize on radiography. In our study, only 19.5% (9/49) of the subjects also had an ultrasound of the knee. Retrospective review of these images showed only one patient with chondrocalcinosis, while the others were unrevealing as cartilage assessment was not directly performed; therefore, further studies would be required to directly compare the sensitivities of these findings on ultrasound and radiography.

One additional explanation for the cartilage icing results in our study relates to the subjects’ serum urate levels, as it has been shown that the sonographic double contour sign disappears when the serum urate falls below 6 ml/dl for 7 months or more [24]. It is unknown if a similar effect can produce false negative evaluation for cartilage icing on radiography. In our subjects with joint aspiration-proven gout, 89% (17/19) had an available serum urate. In the 47% (8/17) of subjects with abnormally elevated serum urate (greater than 6.0 mg/dl in females and 7.8 mg/dl in males), 62.5% (5/8) showed cartilage icing on radiography in contrast to 37.5% (3/8) where cartilage icing was not identified. In the 3 gout subjects with a serum urate below 6 mg/dl, one showed cartilage icing on radiography whereas 2 did not.

### Chondrocalcinosis

Chondrocalcinosis, or calcification of the hyaline cartilage and fibrocartilage, is a finding that is associated with many conditions, including CPPD, diabetes mellitus, degenerative joint disease, gout, hyperparathyroidism, hemochromatosis, and Wilson disease, among others [25]. With CPPD, in addition to the finding of chondrocalcinosis, crystals may also be deposited at other sites, such as synovium, joint capsule, tendons, and ligaments, although typically after articular cartilage involvement [26]. The EULAR agreed that ‘CPPD’ should be the umbrella term that includes acute calcium pyrophosphate (CPP) crystal arthritis, osteoarthritis (OA) with CPPD and chronic CPP crystal inflammatory arthritis [6].

In our study, chondrocalcinosis on radiography was more common in CPPD than gout (50–57% versus 5% sensitivity). When chondrocalcinosis was present, there was an 94% positive predictive value for CPPD versus gout, in comparison to only a 6% positive predictive value for gout versus CPPD. The location of chondrocalcinosis in our study subjects with CPPD was most commonly the meniscus (sensitivity: lateral meniscus 58–64%, posterior meniscus 39–50%, medial meniscus 38–41%, anterior meniscus 30–35%). In no cases (0/49) with CPPD or gout was chondrocalcinosis identified in the hyaline cartilage and not the meniscus; however, the presence of chondrocalcinosis involving the hyaline cartilage at any location had 100% specificity and 100% positive predictive value in the diagnosis of CPPD versus gout.

### Amorphous tophus-like opacities

In our study 5% (1/19) of subjects with gout showed radiographic evidence of a patellar tendon tophus, and 0% (0/30) of subjects with CPPD. The finding of a patellar tendon tophus on radiography was only present in one gout subject. Tophi at the popliteal groove of the femur involving the proximal popliteus tendon have been described on ultrasound in patients with gout [27]; however, in our radiographic study, a popliteus tophus-like opacity was seen in 0% (0/17) of gout subjects and 15% (4/26) of CPPD subjects. Of interest, the presence of a “tophus” with CPPD has so far only been described in case reports [28, 29]. Amorphous tophus-like opacities were also identified in 7% (2/30) of CPPD subjects at other periarticular sites. The etiology of the described “tophi” in this study is unknown given that no direct aspiration of the amorphous opacities was performed. While it is possible that these amorphous areas of increased soft tissue density represent a manifestation of CPPD, these subjects could potentially have CPPD of the joint and gouty tophi deposits outside of the joint, although serum urate was normal in one subject and not available in the second subject. Dual-energy CT was not obtained in our subjects.

### Gastrocnemius tendon calcification

Prior studies have shown gastrocnemius tendon calcification in 21–28% of subjects with chondrocalcinosis [30, 31] and up to 41% when symptomatic [7]. In our study with joint aspiration-proven crystal disease, gastrocnemius calcification on a lateral knee radiograph was seen in 30% (8/27) of CPPD subjects and 5% (1/19) of subjects with gout. Of interest, this latter subject with gout had no chondrocalcinosis and only cartilage icing at the posterior compartment; nonetheless, the presence of gastrocnemius calcification showed 95% specificity and 89% positive predictive value in the diagnosis of CPPD deposition disease versus gout. In the CPPD subjects, gastrocnemius calcification was associated with chondrocalcinosis in 100% (8/8) of patients. However, we cannot exclude the presence of BCP crystals as a possible cause of calcifications.

### Osteoarthritis

Osteoarthritis was more pronounced in patients having CPPD than in patients having gout. Disproportionate narrowing of the patellofemoral joint has been described as a characteristic finding of CPPD [1, 32–35]. In our study, severe patellofemoral osteoarthritis, according to Kellgren-Lawrence [15], was present in 30% (9/30) of CPPD patients; however, only in one subject was this disproportionate to other knee compartments. There was mostly none or mild osteoarthritis in our gout subjects. Interestingly, BCP has been described as being the leading crystals in mineralization of articular cartilage rather than CPPD in patients with osteoarthritis [36]. This stresses out the need for diagnostic accuracy to guide therapeutic strategies.

### Joint effusion

Joint effusion on ultrasound in the presence of gout or CPPD has been described in 35% or 37%, respectively [37]. In our radiographic study, a joint effusion was present at time of imaging in 100% (30/30) of patients having CPPD and in nearly 80% (15/19) of patients with gout. Our higher prevalence may relate to the higher likelihood of joint pathology, as we are assuming that our patients had symptoms or clinical examination findings that warranted radiography and joint fluid aspiration. Selection bias may also have been introduced as a radiographic finding of joint recess distention may more likely result in joint aspiration, which was our standard of reference.

### Limitations

We acknowledge several limitations of our study, which includes the retrospective matter of this study. While the number of patients in our study was limited, this was due to our strict inclusion criterion of only aspiration-proven diagnosis. Thus, the number of patients did not allow to match both groups according to age. Additional radiographic findings (e.g. tophus formation, gastrocnemius tendon calcifications, osteoarthritis, and joint effusion) were evaluated by consensus, which might have introduced a bias due to the different level of experience between both readers. As another limitation, neither sonographic correlation for the double contour sign, nor dual-energy CT to evaluate for monosodium urate depositions were available. Additionally, the mean aspiration time from imaging was 61 days. The effect of this difference on the aspirated crystals is unknown. However, the majority of our patients had a joint aspiration within 4 days of imaging. Other crystal deposition diseases, e.g. BCP, cannot be excluded due to their lack of visibility in light microscopy. Additionally, diagnosis of CPPD using synovial fluid analysis has limitations [38, 39], which could have an influence on our study population. Although the diagnosis of gout or CPPD was proven by synovial fluid analysis, pathologic proof of icing, chondrocalcinosis, tophus, or gastrocnemius calcification was not possible. Dual-energy CT or multi-energy spectral photon-counting CT could have been key factors to analyze the causing crystals, show multi-crystal involvement, and show differential diagnoses, e.g. hydroxyapatite crystal deposits [40–42]. One final limitation is that we did not include a control group of aspiration negative diagnosis, because a high false negative rate for CPPD crystal analysis is known in the literature. This is due to the small crystal size [10, 43]. Thus, an aged matched control group might have included false negative diagnoses of CPPD.

## Conclusion

In subjects with joint aspiration-proven crystal disease of the knee, the radiographic finding of cartilage icing was seen in both gout and CPPD. Chondrocalcinosis (overall and hyaline cartilage) as well as gastrocnemius tendon calcification positively correlated with the diagnosis of CPPD over gout.
